# Deciphering the microbial succession and color formation mechanism of “green-covering and red-heart” *Guanyin Tuqu*

**DOI:** 10.3389/fmicb.2024.1412503

**Published:** 2024-07-23

**Authors:** Liping Zhu, Liang Chen, Bin Lin, Yin Xu, Weiwei Dong, Yijun Lv, Jie Tang, Gang Zhang, Lei Zhang, Shengzhi Yang, Qiang Yang, Shenxi Chen

**Affiliations:** ^1^Hubei Key Laboratory of Quality and Safety of Traditional Chinese Medicine and Health Food, Jing Brand Research Institute, Jing Brand Co., Ltd, Daye, China; ^2^Hubei Key Laboratory of Edible Wild Plants Conservation and Utilization, College of Life Sciences, Hubei Normal University, Huangshi, China

**Keywords:** “green-covering and red-heart” *Guanyin Tuqu*, microbial community, temporal succession, environmental variables, spatial position, color formation

## Abstract

“Green-covering and red-heart” *Guanyin Tuqu* (GRTQ), as a type of special fermentation starter, is characterized by the “green-covering” formed on the surface of *Guanyin Tuqu* (SQ) and the “red-heart” in the center of *Guanyin Tuqu* (CQ). However, the mechanisms that promote temporal succession in the GRTQ microbial ecology and the formation of “green-covering and red-heart” characteristics remain unclear. Herein, we correlated the temporal profiles of microbial community succession with the main environmental variables (temperature, moisture, and acidity) and spatial position (center and surface) in GRTQ throughout fermentation. According to the results of high-throughput sequencing and culture-dependent methods, the microbial communities in the CQ and SQ demonstrated functional complementarity. For instance, the bacterial richness index of the CQ was greater than that of SQ, and the fungal richness index of the SQ was greater than that of CQ at the later stage of fermentation. Furthermore, *Saccharomycopsis*, *Saccharomyces*, *Aspergillus*, *Monascus*, *Lactobacillus*, *Bacillus*, *Rhodanobacter*, and *Chitinophaga* were identified as the dominant microorganisms in the center, while the surface was represented by *Saccharomycopsis*, *Aspergillus*, *Monascus*, *Lactobacillus*, *Acetobacter*, and *Weissella*. By revealing the physiological characteristics of core microorganisms at different spatial positions of GRTQ, such as *Aspergillus clavatus* and *Monascus purpureus*, as well as their interactions with environmental factors, we elucidated the color formation mechanism behind the phenomenon of “green” outside and “red” inside. This study provides fundamental information support for optimizing the production process of GRTQ.

## Introduction

“Green-covering and red-heart” *Guanyin Tuqu* (GRTQ), as a kind of special fermentation starter in the southern provinces of China, plays a vital role in initiating the fermentation of the light-flavor *Baijiu* (Zheng and Han, 2016; Jiang et al., 2018; Zhu et al., 2022). The raw material of GRTQ mainly consisted of “*Guanyin*” clay, rice bran, *Zhongqu* (mother *Tuqu*), and *Monascus* wheat bran, which were manually kneaded into a spherical billet with a diameter of approximately 8 cm. The open manufacturing and fermentation environment of GRTQ provided conditions for the enrichment of microorganisms (Du et al., 2019). During the fermentation process of GRTQ, the green part formed on the surface was called “green-covering,” and the red part formed in the center was called “red-heart.” According to practical production experiences, GRTQ with an appropriate amount of “red-heart” and “green covering” was usually considered to be a reflection of high quality (Li et al., 2010; Hu et al., 2016). Previous study has reported that *Neurospora crassa*, *Aspergillus nidulans*, *Bacillus subtilis*, and *Oceanobacillus iheyensis* were chameleon-like microbes that regulated the metabolic differences of five-member heterocyclic amino acids in three-color sauce-flavor *Daqu*, resulting in microecological differentiation (Yang et al., 2023). Dong et al. (2024) found that *Kroppenstedia*, *Virgibacillus*, and *Bacillus* as dominant bacteria in black *Daqu*, yellow *Daqu*, and white *Daqu* of sauce-flavor *Jiuqu*, severally. However, no in-depth research has been conducted on how the “green-covering and red-heart” coloration arises and how they affect the quality of GRTQ.

To date, the production of GRTQ mainly depends on traditional crafts, which are characterized by low mechanization and rely on the experience of craftsmen to adjust the process parameters, which easily leads to dissimilar communities and qualities among different batches of GRTQ (Han et al., 2023). The quality of *Jiuqu* (a fermentative agent) depends largely on the composition of the microbial community and metabolic characteristics. Microbes can secrete lots of hydrolases, flavor compounds, and functional substances during the fermentation of *Jiuqu* (Wang and Xu, 2015). Thus, it is necessary to obtain a comprehensive understanding of the microbial diversity and function of GRTQ and brewing microbial resources, which is the premise and foundation for fully analyzing the fermentation mechanism of light-flavor *Baijiu* and improving its the product quality. Therefore, a comprehensive understanding of the spatial distribution of microbial composition and microbial succession patterns during *Tuqu* fermentation is required (Jin et al., 2019). Previous studies have reported that environmental factors such as moisture (Lei et al., 2020), temperature (Xiao et al., 2017; Fu et al., 2021; Zhao et al., 2022), pH (Narendranath and Power, 2005), and acidity (Zheng et al., 2018; Hao et al., 2021) were the driving factors of microbial community succession in *Jiuqu*. For example, Ma et al. (2022) reported that temperature, humidity, and acidity were the main driving factors of microbial succession during *Daqu* fermentation. Deng et al. (2020) reported that variations in temperature and moisture led to microbial diversity and various metabolites during spontaneous solid-state fermentation of high-temperature *Daqu*. To our knowledge, the major driving factors for microbial community in *Tuqu* have not been reported. Therefore, it is crucial to study the microbial community succession mechanism and environmental factors affecting microbial growth during the cultivation process of GRTQ to stabilize the quality and establish a modern production workshop for GRTQ.

In this study, samples of GRTQ from different fermentation stages were collected. By high-throughput sequencing combined with a classical culture-dependent approach, the microbial community succession in the samples were statistically analyzed to determine the dynamic changes in microorganisms throughout the production process at the center and on the surface. Furthermore, the correlations between the core microbiota and environmental variables (temperature, moisture, and acidity), as well as the growth characteristics of *Aspergillus clavatus* and *Monascus purpureus* were investigated, revealing the mechanism underlying the “green-covering” and “red-heart” phenomenon. These results could provide reference support for optimizing the production process of GRTQ and establishing modern production lines.

## Materials and methods

### Sample collection

**Figure 1:**
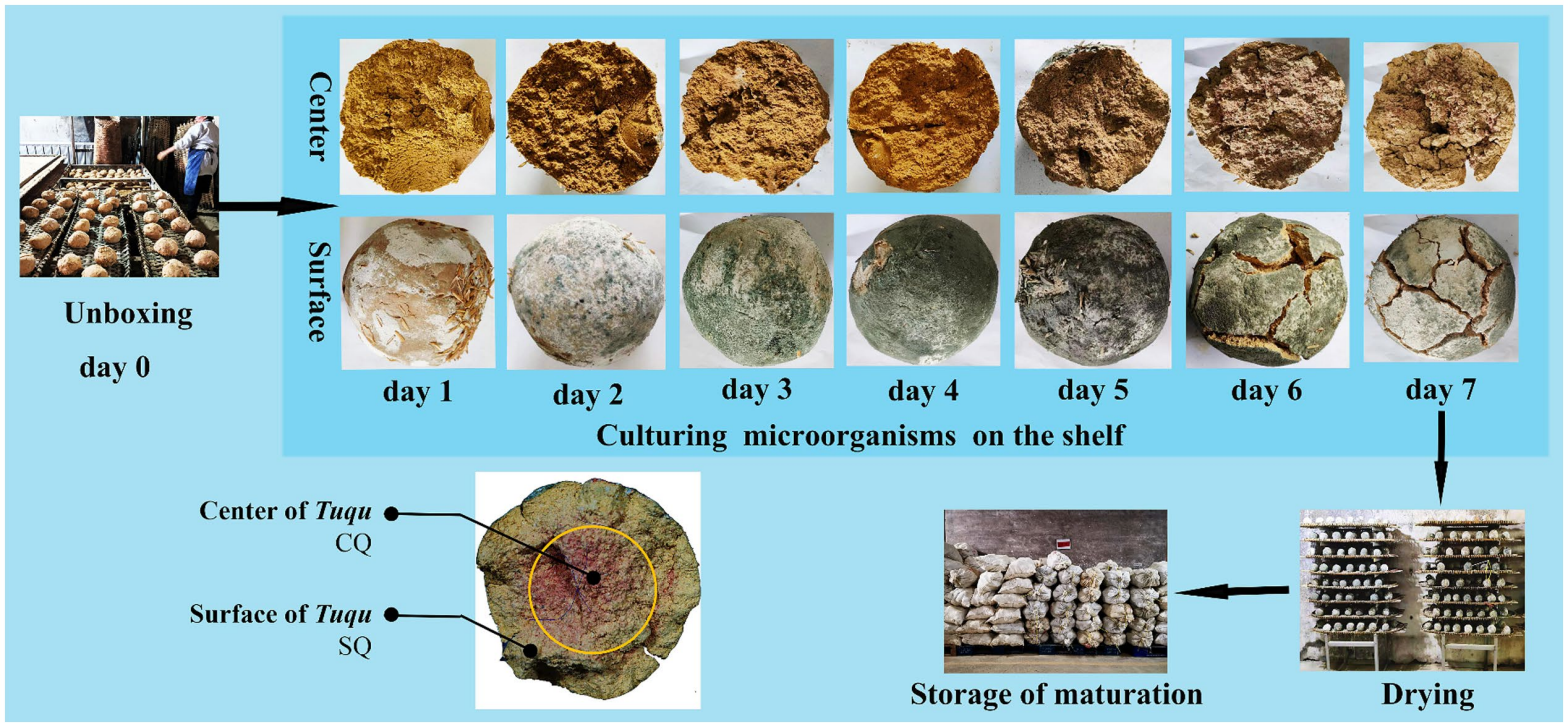
Schematic of the production process of “green-covering and red heart” *Guanyin Tuqu* and the appearance of the center and the surface.

“Green-covering and red-heart” *Guanyin Tuqu* (GRTQ) samples were collected from the *Tuqu*-making workshop in Daye, Hubei province, China. GRTQ was made from the *Guanyin* clay [73.8%, weight (w)], rice bran (24.6%, w), *Zhongqu* (1.6%, w), and *Monascus* wheat bran (1.2%, w), and the water content was kept at 32%. Next, the mixture was manually kneaded into a spherical billet with a diameter of approximately 8 cm. These spherical billets were placed in the box bed for the preculture at 27°C for 23 h termed culture microorganisms. Then, the obtained billets were transferred to the steel shelf in another room for the second incubation, and the temperature of the culture room was controlled at 30°C. After cultivation for 7 days, the culture room was maintained at 35°C to reduce the water content of GRTQ to <10%, which was called the dry period. The selected samples included: GRTQ cultured microorganisms, GRTQ fermented for days 1–7, and drying stage, which were labeled as 0, 1, 2, 3, 4, 5, 6, 7, and D, respectively. Each GRTQ sample was divided into two parts, included the surface layer of 1.5 cm thick was named surface part of *Tuqu* labeled as SQ, and the remaining central part was named central part of *Tuqu* labeled as CQ (Figure 1). Each sample had three biological replicates. A total of 54 samples were obtained. These samples were stored at 4°C until further analysis, and −20°C for DNA extraction.

### Physicochemical properties

The moisture content, total acids, and temperature of GRTQ samples were determined. After drying at 150°C for 90 min, the moisture content of the GRTQ powder was calculated using the gravimetric method. Total acids was determined by titration with 0.1 M NaOH and 100 μL of phenolphthalein to obtain a titration endpoint of pH 8.2. The temperature was measured by inserting the thermometer directly into the center of the *Tuqu*.

### Isolation and enumeration of different culturable microbes

Ten grams of GRTQ powder were homogenized in 90 mL of sterile water, and incubated for 25 min continuously stirred at 160 rpm under 20°C. This suspension was defined as a 1/10 dilution and was serially diluted 10-fold with sterile water. A 100 μL aliquot of each dilution was spread and incubated on the agar media (showed in Table 1; Rosfarizan and Ariff, 2000; Shi et al., 2006; Zhou et al., 2021). The media for culturing fungi and bacteria were supplemented with final concentrations of 0.5 g/L chloramphenicol and 0.5 g/L nystatin, respectively. The enumerations of each group of microbes for each sample were performed in triplicate. The above reagents were purchased from Solarbio Science & Technology Co., Ltd., Beijing, China.

**Table 1:** Isolation materials and methods for different culturable microorganisms.

Microbial species	Media component	Separation principle	Culture conditions
Yeasts	Yeast extract peptone dextrose agar (YPD): 10 g/L yeast extract, 20 g/L peptone, 20 g/L glucose, and 20 g/L agar.	Peptone and yeast extract powder provide carbon and nitrogen sources, and glucose provides fermentable sugars for yeasts.	30°C for 72 h
Aerobic bacteria	Wallerstein nutrient agar (WL): 5 g/L casein peptone, 4 g/L yeast extract, 50 g/L glucose, 0.425 g/L KCl, 0.125 g/L CaCl_2_, 0.125 g/L MgSO_4_, 0.0025 g/L FeCl_3_, 0.0025 g/L MnSO_4_, 0.55 g/L KH_2_PO_4_, 0.022 g/L bromocresol green, 20 g/L agar, and pH 5.5.	KCl, CaCl_2_, and FeCl_3_ are critical ions and help maintain osmotic balance.	37°C for 48 h
Molds and *Aspergillus clavatus*	Potato dextrose agar (PDA): 6 g/L potato infusion powder, 20 g/L glucose, 20 g/L agar, and pH 5.6.	Potato extract is beneficial to the growth of all kinds of molds, and glucose can provide carbon source.	30°C for 72 h
*Lactobacillus*	De Man, Rogosa, and Sharpe agar (MRS): 10 g/L peptone, 8 g/L beef extract, 4 g/L yeast extract, 20 g/L glucose, 2 g/L KH_2_PO_4_, 2 g/L ammonium citrate dibasic, 5 g/L CH_3_COONa, 0.2 g/L MgSO_4_, 0.04 g/L MnSO_4_, 1 g/L Tween-80, 20 g/L agar, and pH 6.5.	Ammonium citrate dibasic, CH_3_COONa, MgSO_4_, MnSO_4_, and tween-80 provide growth factors for the culture of various *Lactobacillus*.	37°C for 48 h
*Monascus*	PDA medium, and 0.2 mL of sterilized lactic acid solution (50%, V:V) was added to cover the sample.	*Monascus* likes lactic acid, and high concentration of lactic acid can inhibit the growth of miscellaneous bacteria.	30°C for 72 h

### The growth characteristics of *A. clavatus* and *M. purpureus*

Preculture conditions: strain was inoculated on a PDA plate at 30°C, pH 6.0 and cultured for 5 days on the aerobic incubator. Regulated batch cultures: the effects of PDA medium at pH (3.07, 4.02, 5.00, 6.09, and 6.97), temperature (25, 30, 35, 40, and 45°C), and oxygen (aerobic incubator and anaeropack box) on the growth of *M. purpureus* and *A. clavatus* were investigated, respectively. Anaerobic box was used to generate anaerobic conditions.

### DNA extraction, amplification, and sequencing

Total genomic DNA of samples was extracted with the TGuide S96 Magnetic Soil/Stool DNA Kit [Tiangen Biotech (Beijing) Co., Ltd.] according to manufacturer’s instructions. The DNA concentration of the samples was measured with the Qubit dsDNA HS Assay Kit and Qubit 4.0 Fluorometer (Invitrogen, Thermo Fisher Scientific, Oregon, United States). The 27F: AGRGTTTGATYNTGGCTCAG and 1492R: TASGGHTACCTTGTTASGACTT universal primer set was used to amplify the full-length 16S rDNA gene, and the 1F: 5′-CTTGGTCATTTAGAGGAAGTAA-3′ and 4R: 5′-TCCTCCGCTTATTGATATGC-3′ universal primer set was used to amplify the ITS rDNA gene from the genomic DNA, extracted from each sample. All PCR reactions were carried out in 20 μL reaction system containing 0.5 mM of each primer, 10 ng of template DNA, and the KOO One PCR Master Mix (TOYOBOLife Science) was used to perform 25 cycles of PCR amplification, the PCR conditions of 16S rDNA region amplifcation were as follows: pre-denaturation at 95°C for 2 min, followed by 25 cycles of denaturation at 98°C for 10 s, annealing at 55°C for 30 s, and extension at 72°C for 1 min 30 s, and a final extension at 72°C for 2 min. The PCR conditions of ITS rDNA region amplifcation were as follows: pre-denaturation at 95°C for 5 min, followed by 25 cycles of denaturation at 98°C for 30 s, annealing at 55°C for 30 s, and extension at 72°C for 45 s, and a final extension at 72°C for 10 min. The total of PCR amplicons were purified with Agencourt AMPure XP Beads (Beckman Coulter, Indianapolis, IN) and quantified using the Qubit dsDNA HS Assay Kit and Qubit 4.0 Fluorometer (Invitrogen, Thermo Fisher Scientific, Oregon, United States). After the individual quantification step, amplicons were pooled in equal amounts. SMRTbell libraries were prepared from the amplified DNA by SMRTbell Express Template Prep Kit 2.0 according to the manufacturer’s instructions (Pacific Biosciences). Purified SMRTbell libraries from the pooled and barcoded samples were sequenced on a single PacBio Sequel II 8 M cell using the Sequel II Sequencing kit 2.0. Raw data FASTQ files were imported into the format which could be operated by QIIME2 system using QIIME tools import program. Demultiplexed sequences from each sample were quality filtered and trimmed, de-noised, merged, and then the chimeric sequences were identified and removed using the QIIME2 dada2 plugin to obtain the feature table of amplicon sequence variant (ASV). The QIIME2 feature-classifier plugin was then used to align ASV sequences to a pre-trained GREENGENES 13_8 99% database to generate the taxonomy table (Bokulich et al., 2018). Any contaminating mitochondrial and chloroplast sequences were filtered using the QIIME2 feature-table plugin. Sequencing service and data analysis service were provided by Wekemo Tech Group Co., Ltd. Shenzhen China. For the sequence data, they were deposited in the national center of biotechnology information (NCBI) database with accession number: PRJNA1080012.

### Statistical analysis

Origin Pro 2018 was used to draw the histogram and line chart. Spearman’s correlation heat map analysis was performed to explore the correlations between environmental variables and microorganisms using the “pheatmap” packages of R (version 4.0.3). Significant differences were tested using one-way ANOVA in SPSS. The microbial communities in surface and center of GRTQ were further compared using the linear discriminant analysis effect size (LEfSe) analysis, Venn diagram and PCoA analysis with the online interface using the Wekemo bioincloud: https://www.bioincloud.tech/standalone-task-ui/lefse.

## Results

### Diversity of microbial community in the fermentation process of GRTQ

**Figure 2:**
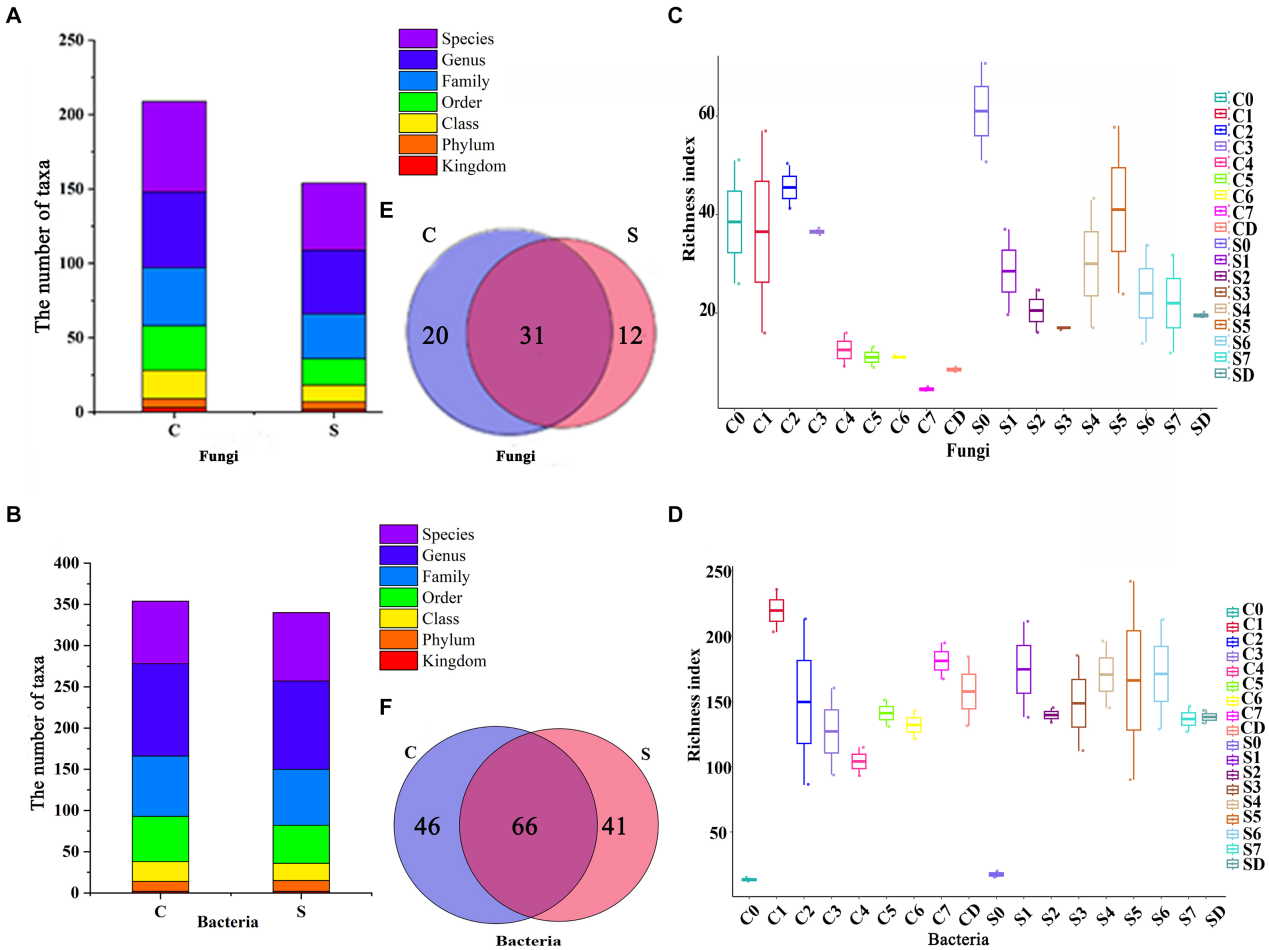
Alpha diversity and the microbial community composition of center and surface. Diagram for the **(A)** fungi and **(B)** bacteria from phylum to genus level. **(C)** Fungal and **(D)** bacterial diversity in terms of richness index at different fermentation times. The lines inside the boxes denote the median at 95% confidence. Venn diagram of **(E)** fungi and **(F)** bacteria according to the genus level. C, Center; S, Surface, 0–7, Days 0–7; D, drying.

After filtering the raw sequencing data by QIIME 2, 152,683 bacterial reads and 138,708 fungal reads from the surface parts of *Tuqu* (SQ) and 164,628 bacterial reads and 161,582 fungal reads from the central parts of *Tuqu* (CQ) were obtained. These bacterial and fungal reads were clustered into 3,808 and 525 OTUs, respectively. The total sequences belonged to 16 phyla and 153 genera of bacteria and 7 phyla and 63 genera of fungi. Among them, 5 phyla and 43 genera of fungi were detected in the SQ. On the other hand, the fungi in the CQ were identified as belonging to 6 phyla and 51 genera (Figure 2A). In addition, 13 phyla and 107 genera of bacteria were detected in the SQ, while the bacteria in the CQ were identified as 12 phyla and 112 genera (Figure 2B). The results indicated that both the SQ and the CQ were inhabited by a quantity of microbial populations, which might indicate that they are highly important for the production of *Baijiu* (Chen et al., 2021).

For alpha diversity, a greater richness index indicated greater community diversity. The fungal richness index of the CQ first increase from 38.5 (Day 0) to 45.5 (Day 2), and then decreased to 8.5 during the drying process (Figure 2C). However, the bacterial richness of the CQ presented an opposite trend to that of fungi, which first decreased from 219.5 (Day 0) to 104 (Day 4), and then raised to 157.5 during the drying process (Figure 2D). The richness index of fungi changed from 61 (Day 0) to 17 (Day 3), then gradually raised to 41 (Day 5), and finally decreased to 19.5 during the drying process in the SQ (Figure 2C). However, there was no obvious change in the richness index of bacteria in the SQ in a narrow range, fluctuating between 130 and 170 (Figure 2D). The richness index of bacteria was far greater than that of fungi both in the SQ and CQ, which suggested that the diversity of bacteria was greater than that of fungi. After Day 4, the fungal richness index of the SQ was greater than that of the CQ, indicating the SQ had more fungal diversity, which might be related to the oxygen content of the SQ (Mo et al., 2022). At the later stage of fermentation (After Day 7), the bacterial richness index of the CQ was greater than that of the SQ, indicating that the CQ had greater bacterial diversity.

We then investigated the number of common and unique genera of the CQ and the SQ during the succession process via a Venn diagram. There were 20 and 12 unique fungal genera in the CQ and SQ, respectively, while there were 46 and 41 unique bacterial genera, respectively (Figures 2E,F), suggesting that the CQ possessed slightly higher uniqueness than the SQ.

### Microbial composition and community succession

**Figure 3:**
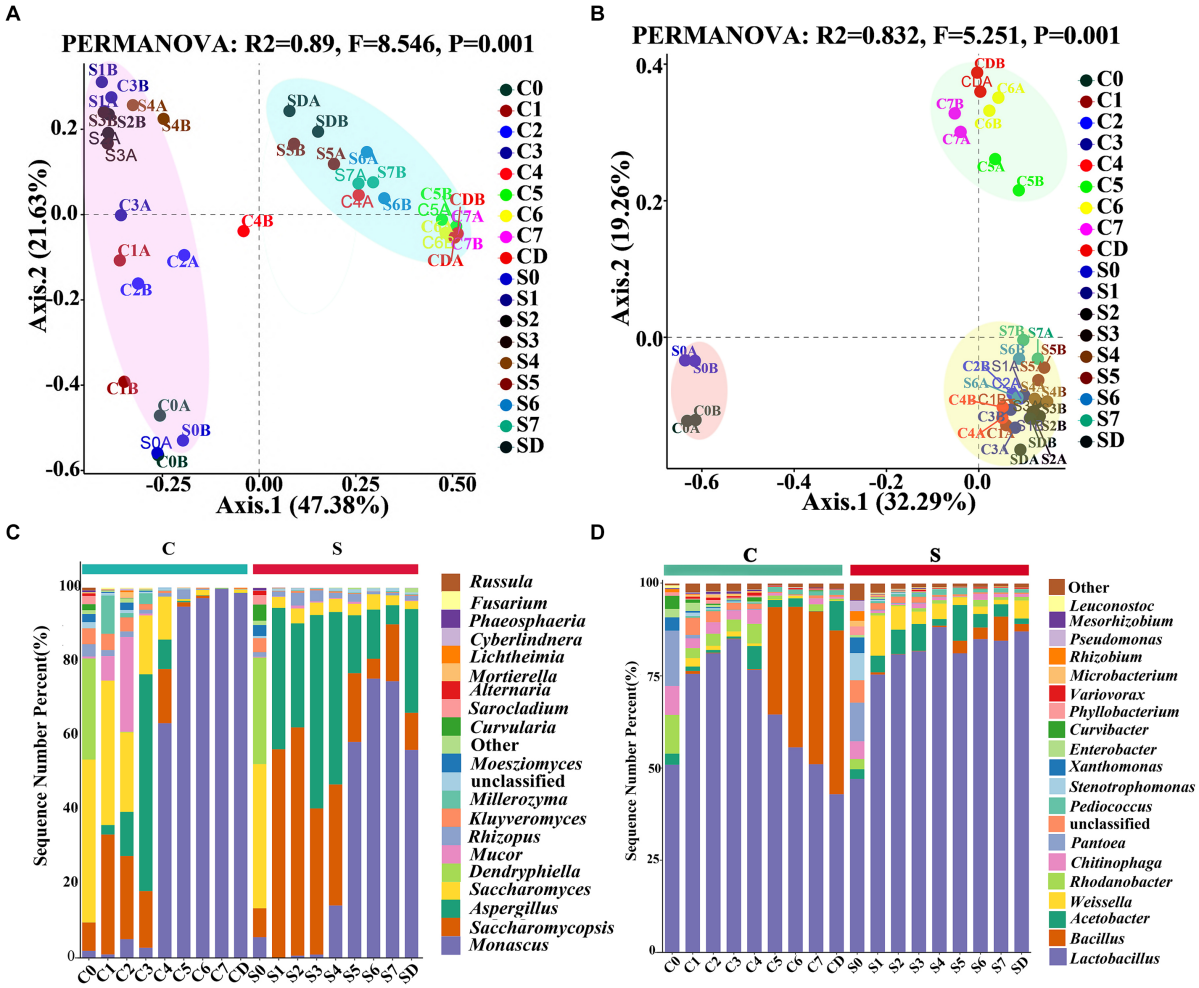
PCoA analysis of the microbial communities associated with the *Tuqu* fermentation process. **(A)** Fungi and **(B)** bacteria based on Bray-Curtis distance. **(C)** Fungal and **(D)** bacterial community compositions and succession at the genus level. Among the top 20 genera with relative abundance were greater than 0.1%. C, Center; S, Surface; 0–7, Days 0–7; D, drying.

PCoA analysis based on Bray–Curtis dissimilarity distances revealed that both bacterial and fungal communities between the CQ and the SQ on different days were clearly separated. The succession process of fungi could be divided into phase 1 (C0-C3 and S0-S4) and phase 2 (C4-CD and S5-SD) throughout the production of GRTQ (Figure 3A). In addition, the bacteria were classified as phase 1 (C0 and S0), phase 2 (C1-C4 and S1-SD), and the subsequent fermentation of the CQ (C5-CD) formed an independent phase 3 (Figure 3B). These results indicated that the successional rhythms of the fungal communities in the CQ and SQ were basically similar, while there were significant differences in the bacterial communities. Specifically, the bacterial communities in the CQ exhibited more intense succession during the later fermentation stage (After Day 4).

The relative abundances and genus compositions of dominant microorganisms were investigated between the CQ and SQ on different days during the fermentation process (Figures 3C,D). Among the top 20 genera with relative abundances were greater than 0.1%, *Saccharomycopsis*, *Saccharomyces*, *Aspergillus*, *Monascus*, *Lactobacillus*, *Bacillus*, *Rhodanobacter*, and *Chitinophaga* were identified as the dominant microorganisms in the CQ, while those in the SQ were represented by *Saccharomycopsis*, *Aspergillus*, *Monascus*, *Lactobacillus*, *Acetobacter*, and *Weissella. Mortierella* only appeared in the CQ, while *Beauveria* only existed in the SQ. During the C0 and S0 periods, the relative abundances of dominant microorganisms were relatively high, and the species and quantities of microorganisms were largely similar, mainly due to the straw curtains covered the GRTQ samples at the beginning of fermentation, providing heat insulation and moisture preservation. Thus, there were few differences in the environments of the SQ and CQ. As fermentation progresses, fungal community succession began to occur. The abundance of *Saccharomycopsis* first increased and then decreased in CQ and SQ. The abundance of *Saccharomycopsis* in the SQ was higher than that in the CQ. At the S2 stage, the maximum proportion of *Saccharomycopsis* was 61.66%, after which the percentage decreased slowly until the end of fermentation. The highest percentage of *Saccharomycopsis* was 32.41% at the C1 stage but distinctly decreased to 1.19% at the C5 stage. Notably, *Saccharomyces*, as dominant yeast in the CQ, was much greater at the early stage of fermentation than at the end of fermentation. Additionally, *Saccharomyces* in the CQ reduced gradually from 44.03% in the C0 period to 0.50% in the C5 period. However, *Saccharomyces* was 38.96% at the initial S0 stage in the SQ, and its abundance was relatively low (< 4%) at the other stages. In the preliminary stage of the SQ (S1-S4), *Aspergillus* was more dominant (> 30%), then gradually decreased with the fermentation process, but it was still higher than 10%. However, *Aspergillus* predominated at the C3 stage (58.58%) and was relatively rare at other stages (< 1%). *Monascus* accounted for a small proportion in the CQ and the SQ during the initial stage of fermentation, but quickly increased from 63.38 to 99.67% in C4-CD, and rising from 58.32 to 75.43% in S5-SD.

We further investigated the distribution and succession of the core bacteria during the GRTQ fermentation process (Figure 3D). The bacterial genus *Lactobacillus* was significantly prevalent at both the CQ (42.87–84.92%) and the SQ (47.05–88.18%). *Bacillus* was present mainly in the late stage of fermentation in the CQ, and multiplied quickly from the C5 to CD stage (29.12–44.43%), which might be due to its ability to withstand high temperature and low water activity in the late stage of fermentation (Zhang et al., 2021). *Acetobacter* mainly existed in the SQ, accounting for 9.67–1.48% (S5-SD). Similarly, *Weissella* also mainly presented in the SQ, occupying for 10.89–1.21% (S1–S7). This is mainly because their growth requires more oxygen. *Rhodanobacter* and *Chitinophaga* grew in the early stage of fermentation (C0-C4), accounting for 2.68–10.52% and 2.67–7.83%, respectively.

**Figure 4:**
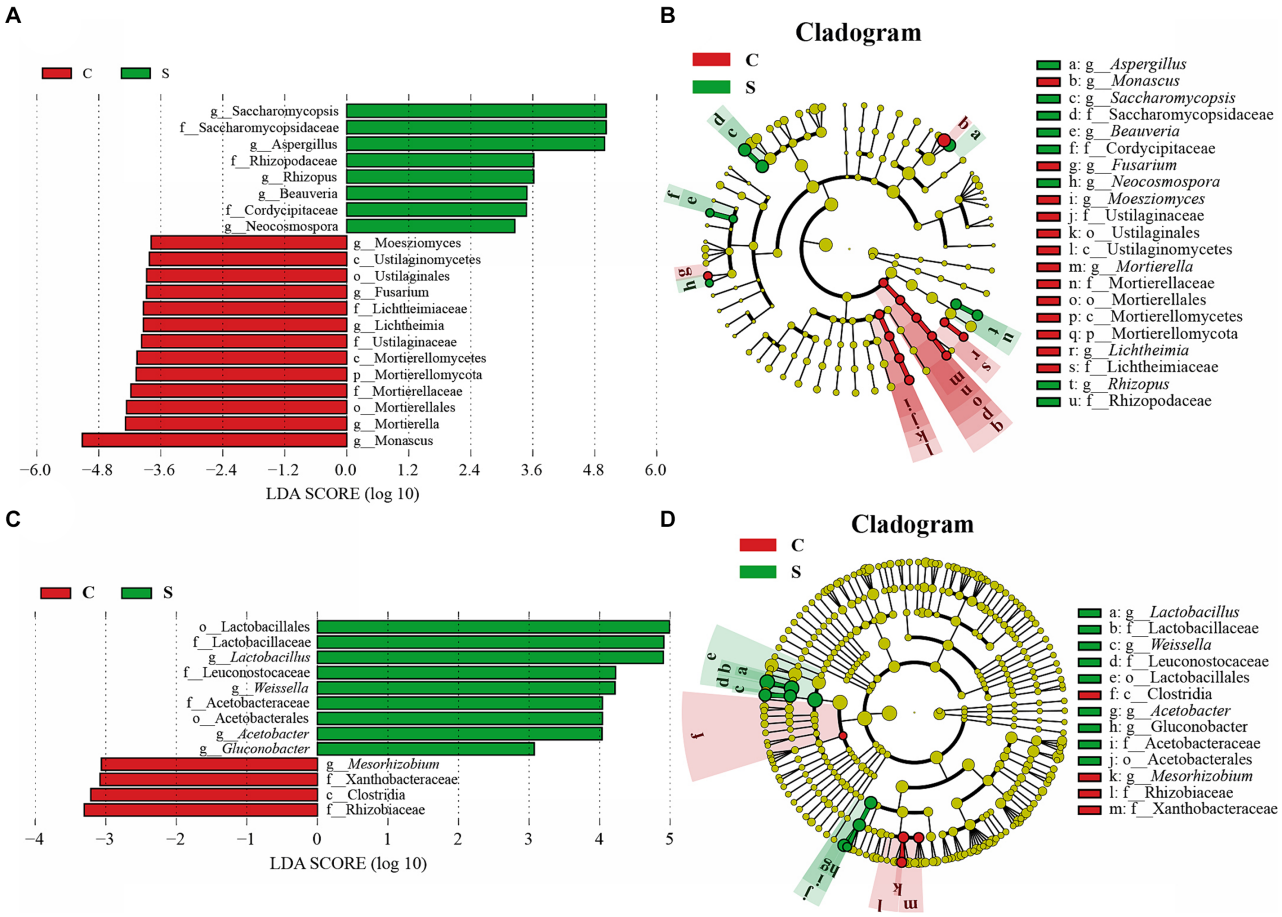
Linear discriminant analysis (LDA) effect size (LEfSe) of the central of *Tuqu* (CQ) and the surface of *Tuqu* (SQ) based on genus level (LDA > 3, *p* < 0.05). The LDA score indicates the level of differentiation between CQ and SQ, and the horizontal bar chart showing discriminant taxa of the fungi **(A)** and bacteria **(C)**. Cladogram of the fungi **(B)** and bacteria **(D)**. Significant discriminant taxon nodes of CQ and SQ are represented by green and red, respectively, while no discriminant taxon nodes are represented by yellow. C, Center; S, Surface.

Because there was little difference in microbial abundance between the CQ and SQ on Day 0 (Figures 3C,D), the abundances of the detected fungal and bacterial taxa in the CQ and SQ samples (excluding Day 0) were further analyzed using the linear discriminant analysis (LDA) effect size (LEfSe) method. Notably, *Saccharomycopsis*, *Aspergillus*, *Rhizopus*, *Beauveria*, and *Neocosmospora* had statistical differences and were distinctive fungal genera in the SQ, while *Monascus*, *Mortierella*, *Lichtheimia*, *Fusarium*, and *Moesziomyces* were statistically different and distinctive fungal genera in the CQ (Figure 4A). Additionally, *Lactobacillus*, *Weissella*, *Acetobacter,* and *Gluconobacter* were discriminant bacterial taxa in the SQ, while *Mesorhizobium* was a discriminant bacterial taxon in the CQ (Figure 4C).

### Enumeration of representative bacteria and fungi

**Figure 5:**
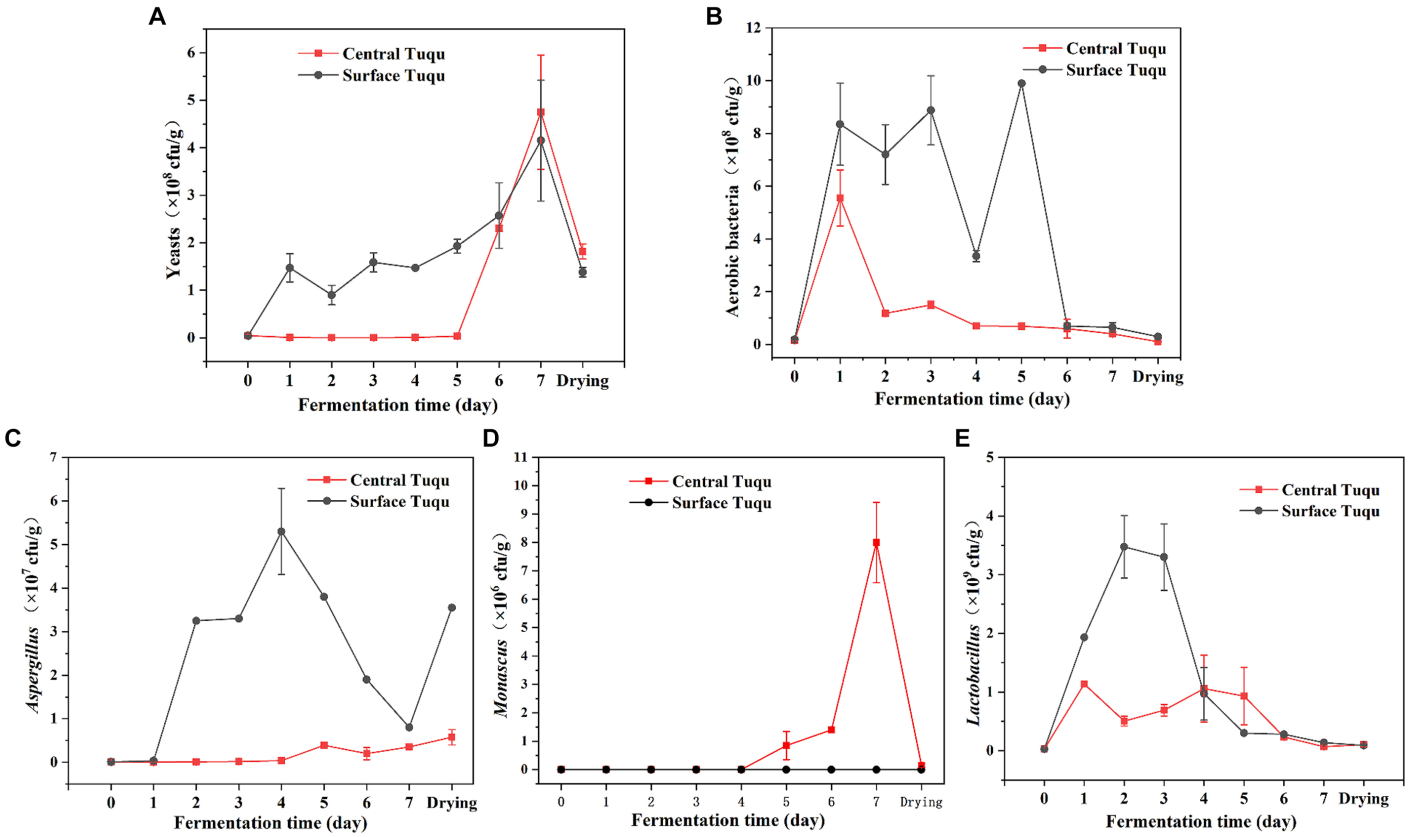
Dynamics of the biomass of different groups of microbes **(A)** yeasts, **(B)** aerobic bacteria, **(C)** *Aspergillus*, **(D)** *Monascus*, and **(E)** *Lactobacillus*, during the *Tuqu* fermentation process. Values are presented as the mean ± SEM.

LEfSe analysis revealed that *Aspergillus*, *Lactobacillus*, and *Monascus* were significant discriminant taxa (Figure 4). Furthermore, through high-throughput sequencing, it was found that *Lactobacillus*, *Monascus,* and *Aspergillus* accounted for a large proportion and played an important role in the fermentation of GRTQ (Figures 3C,D). Therefore, the dominant microorganisms (viable yeasts, aerobic bacteria, *Lactobacillus*, *Aspergillus*, and *Monascus*) were quantitatively investigated using the plate-counting method throughout the fermentation process of the SQ and the CQ. As shown in Figure 5, yeasts proliferated extensively in the later stage of fermentation in GRTQ, with a higher number of yeasts in the SQ than in the CQ. *Aspergillus* was mainly distributed in the SQ, and its biomass peaked on Day 3, followed by a continuous decline. *Monascus* was mainly detected in the CQ and reached its maximum quantity at the end of fermentation (Day 6). Aerobic bacteria and *Lactobacillus* increased rapidly in the first 2 days and followed by a decrease in biomass.

### Temporal changes in the environmental variables

**Figure 6:**
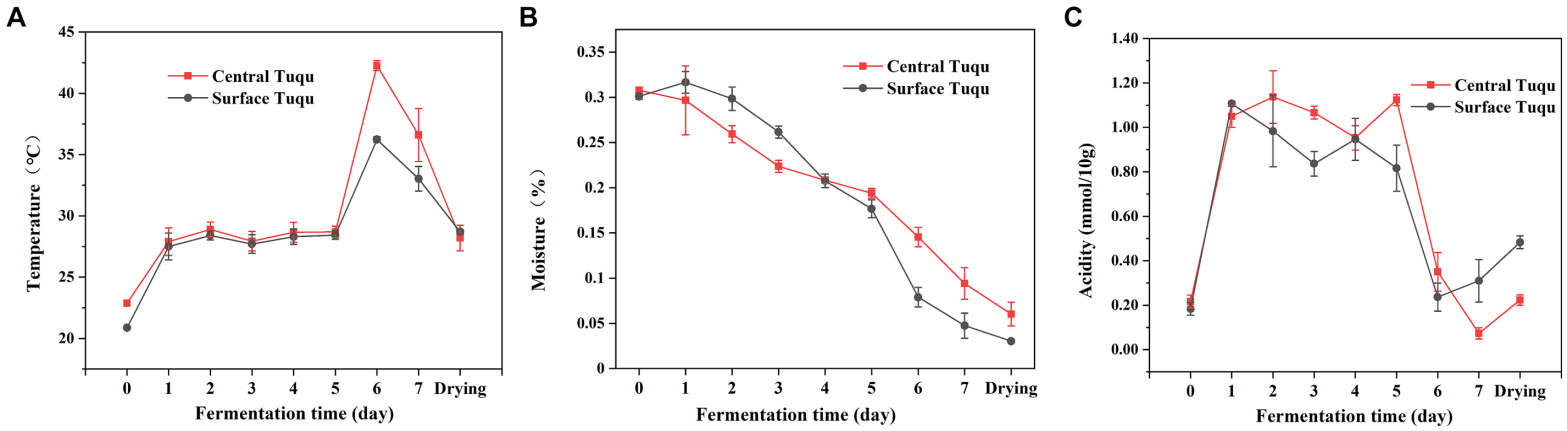
Dynamics of physicochemical characteristics **(A)** temperature, **(B)** moisture, **(C)** acidity during the *Tuqu* fermentation process. Values are presented as the mean ± SEM.

The dynamics of the environmental variables throughout the fermentation process were shown in Figure 6. Temperature is an important indicator of GRTQ fermentation and a valuable factor for screening functional microorganisms of GRTQ (Wang and Xu, 2019). When the prepared GRTQ entered the culture room, its temperature gradually increased and then plateaued for a few days (Day 1–5). After the 5th day, with the rapid reproduction of microorganisms, the temperature of GRTQ rapidly increased and reached its highest value (approximately 45°C) on the 6th day. At the end of cultivation, the microorganisms tended to stabilize, and the temperature of the GRTQ decreased to around 40°C. In the early stage of cultivation, the temperatures of CQ and SQ were similar. However, in the rapid heating phase (Day 5–6), the temperature of the CQ was significantly higher than that of the SQ until the end of cultivation (Figure 6A). The overall moisture content of the GRTQ exhibited a continuous downward trend, ranging from 32 to 3%. Before the 4th day, the moisture of the CQ was lower than that of the SQ, while after the 4th day, the opposite trend was observed. In the early stage, due to the high ambient humidity, the moisture increased slightly. In the later stage, owing to the increase in temperature, the internal water retention of GRTQ was better and its water evaporation was less. Therefore, the moisture content of the CQ was higher than the SQ (Figure 6B). The acidity of the GRTQ first increased, then fluctuated, and finally decreased. The acidity of the CQ was greater than that of the SQ on Days 1–6, and the situation reversed in the later period (Day 7-drying process) (Figure 6C).

### Correlations between environmental variables and microbial community

**Figure 7:**
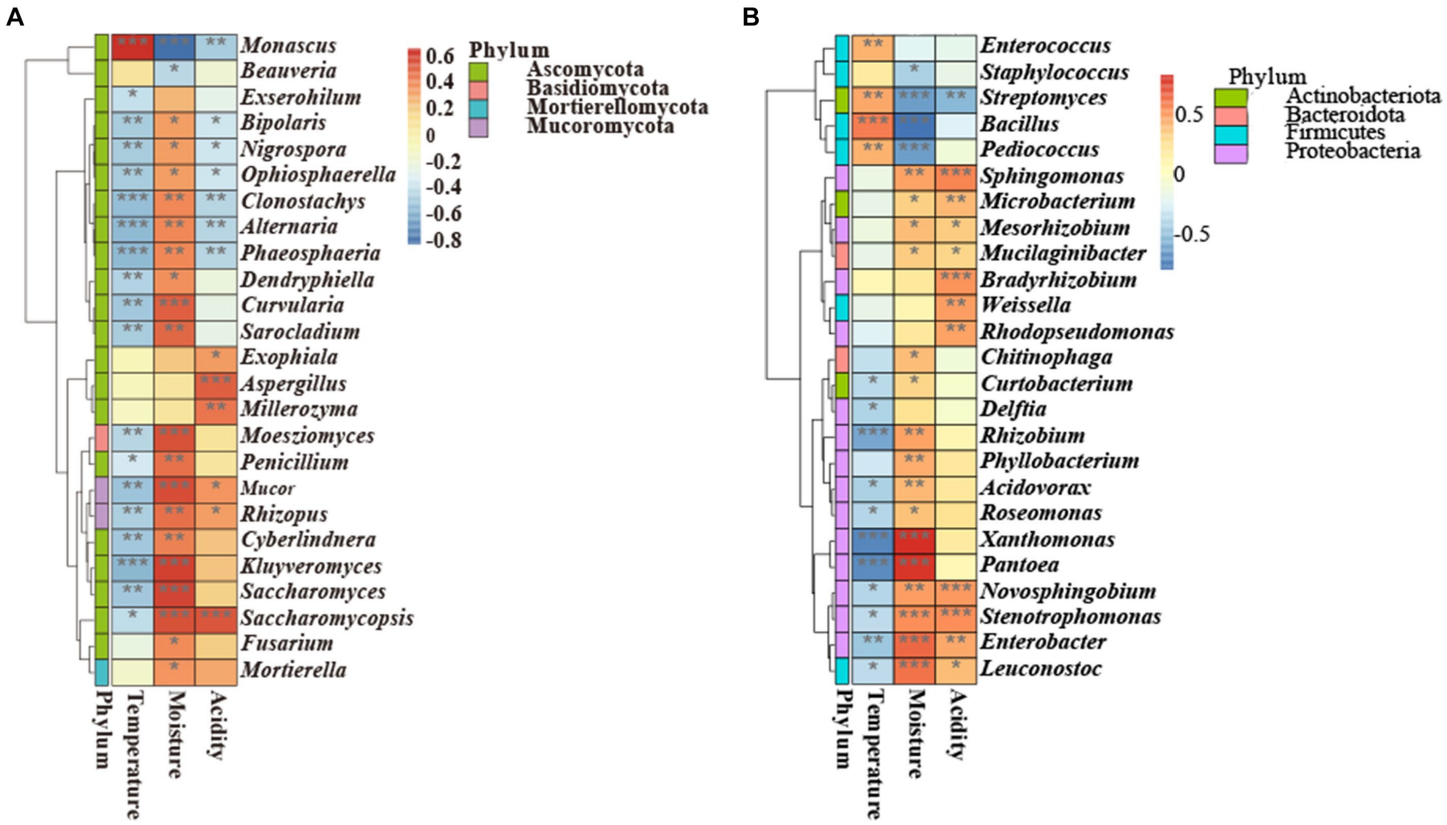
Correlation heatmap analysis of the **(A)** fungi and **(B)** bacteria with environmental factors. With red standing for a perfect positive relationship and blue for a perfect negative correlation. *0.01≤ *p* < 0.05, **0.001≤ *p* < 0.01, *** *p* < 0.001.

There was a correlation between environmental variables (temperature, moisture, and acidity) and microorganisms at the genus level (Figure 7). Specifically, *Monascus* was positively correlated with temperature (*p* < 0.001) and negatively correlated with moisture (*p <* 0.05) and acidity (*p <* 0.01). Moreover, *Saccharomycopsis* was negatively correlated with temperature (*p <* 0.05) and positively correlated with moisture (*p <* 0.001) and acidity (*p <* 0.001). These results demonstrated that the changes in temperature, moisture, and acidity had opposite effects on the growth of *Saccharomycopsis* and *Monascus*. *Saccharomyces* was negatively correlated with temperature (*p* < 0.01) and positively correlated with moisture (*p* < 0.001). *Bacillus* was positively correlated with temperature (*p <* 0.001) and negatively correlated with moisture (*p <* 0.001). *Aspergillus* was positively correlated with acidity (*p* < 0.001). Additionally, *Phaeosphaeria*, *Alternaria*, *Clonostachys*, *Ophiosphaerella*, *Nigrospora*, and *Bipolaris* were negatively related to temperature and acidity, illustrating that the fermentation of GRTQ could inhibit undesirable microbes (Licandro et al., 2020; Zhou et al., 2021; Dong et al., 2022).

### Study on the growth characteristics of *A. clavatus* and *M. purpureus*

**Figure 8:**
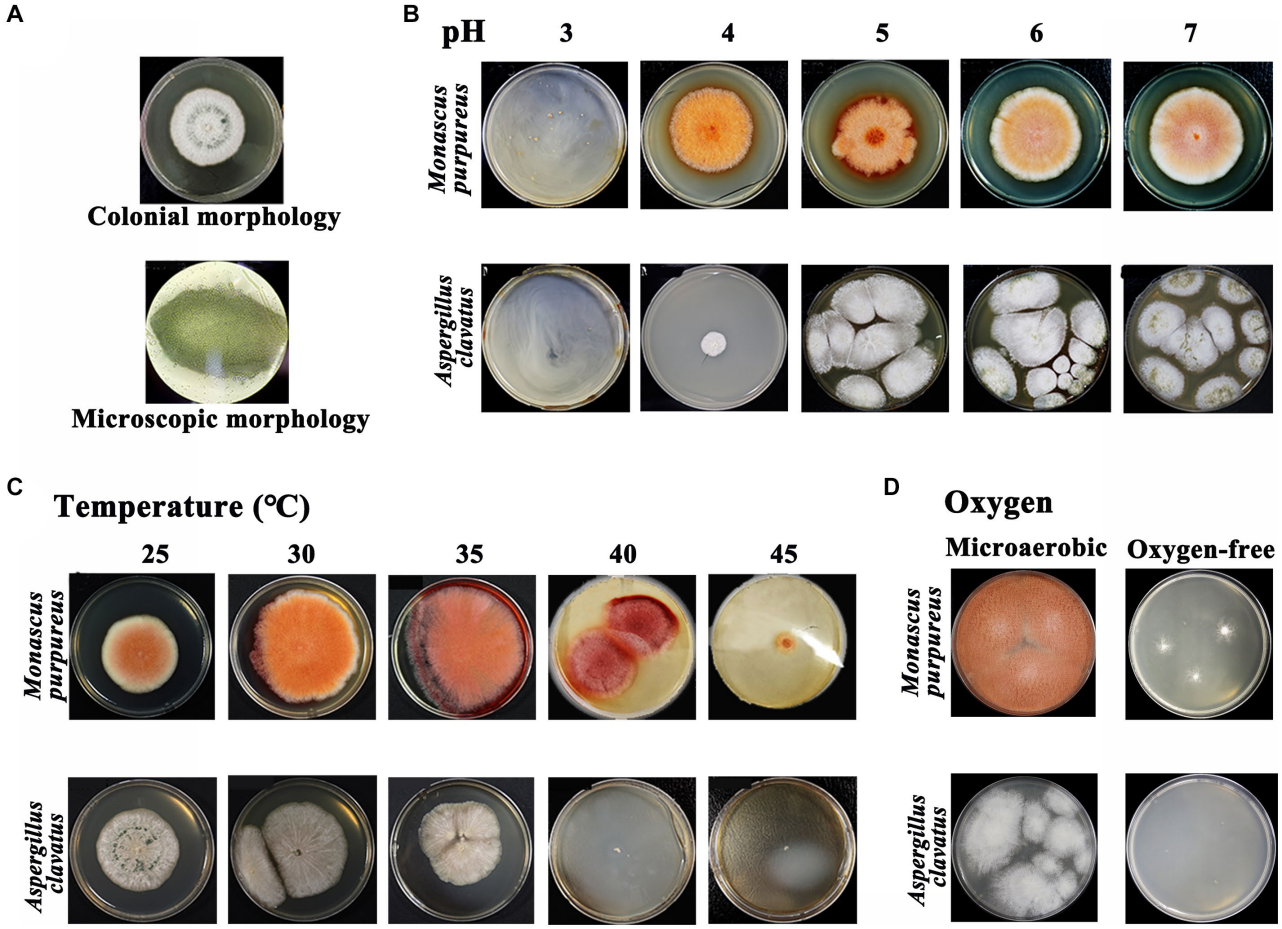
**(A)** Colonial morphology and microscopic morphology (×100) of *Aspergillus clavatus.* Morphology of *Monascus* sp. and *Aspergillus clavatus* at different levels under various environmental factors: **(B)** pH, **(C)** temperature, and **(D)** oxygen. *Monascus* sp.: *Monascus purpureus* was selected as the experimental object.

The apparent characteristics of GRTQ during the fermentation process were shown in Figure 1. The “green-covering” was found to be produced by the growth of *A. clavatus* through separation, screening, and identification experiments (Figure 8A), however, we did not isolate any other microbes that produced green colonies on the green surface of GRTQ, except for *A. clavatus*. On the other hand, according to the previous research in our laboratory, the “red-heart” was generated by the metabolism of *Monascus* sp. (*M. purpureus* and *Monascus ruber*) (Zhu et al., 2022). These results were consistent with the identification of *A. clavatus* and *M. purpureus* as significant discriminant taxa in the SQ and CQ samples, respectively, based on LEfSe analysis at species level (Supplementary Figure S1).

The physicochemical properties of GRTQ play an important role in the growth of microorganisms, providing indispensable support for microbial physiological activity. Therefore, the physiological characteristics of *Monascus* sp. (*M. purpureus* was selected as the experimental object) and *A. clavatus* under the different environmental conditions were investigated to determine the reasons for the “green-covering” and “red-heart” phenomena.

Acidity is one of the essential factors affecting microbial growth, and the results were shown in Figure 8B. When the pH was <3.07, the growth of *M. purpureus* was inhibited (Zhou et al., 2021). However, the growth of *A. clavatus* was inhibited when the pH was <4.02, which indicated that the acid resistance of *M. purpureus* was greater than that of *A. clavatus*. In addition, the optimum growth temperatures for *M. purpureus* and *A. clavatus* were 35 and 30°C, respectively (Figure 8C). The growth of *A. clavatus* was inhibited at temperatures >40°C. Similarly, it also had an inhibitory effect on *M. purpureus* when the temperature was above 45°C, and *M. purpureus* could still grow slowly. This result might indicated that *M. purpureus* was more resistant to high temperature than was *A. clavatus*. The oxygen contents in the SQ and the CQ were obviously different, which might affect the growth of microorganisms (Cui, 2007). Therefore, the tolerances of *M. purpureus* and *A. clavatus* to microaerobic or even anaerobic conditions were analyzed. As shown in Figure 8D, in a microaerobic environment, *A. clavatus* grew slowly without green spores, while *M. purpureus* grew normally. In an oxygen-free environment, *A. clavatus* stopped growing, while *M. purpureus* grew slowly but did not produce pigments. *M. purpureus* had better tolerance to hypoxia and better adapt to the hypoxic environment of the CQ, while *A. clavatus* had a stronger dependence on oxygen and was more suitable for the environment of the SQ.

## Discussion

“Green-covering and red-heart” *Guanyin Tuqu* (GRTQ), is a special fermentative starter (also known as *Jiuqu* in Chinese) that originated in southern China and is characterized by a layer of green covering the surface and with a red heart. It plays a vital role in the production of light-aroma-type *Baijiu*. The GRTQ with a green covering and red heart is often considered to be of high quality. However, the mechanisms that promote temporal succession in the GRTQ microbial ecology and the formation of a “green covering and red heart” remain unclear. Therefore, we correlated the temporal profiles of microbial community succession with the main environmental variables (temperature, moisture, and pH) and spatial position (center and surface) in GRTQ throughout fermentation to reveal the underlying mechanisms involved.

For the microbial community of the whole GRTQ, *Saccharomycopsis*, *Saccharomyces*, *Aspergillus*, *Monascus*, *Lactobacillus*, *Bacillus*, *Rhodanobacter*, and *Chitinophaga* were identified as the dominant microorganisms in the CQ, while the SQ was represented by *Saccharomycopsis*, *Aspergillus*, *Monascus*, *Lactobacillus*, *Acetobacter*, and *Weissella*. *Saccharomycopsis*, *Saccharomyces*, *Aspergillus*, *Monascus*, and *Lactobacillus* were common dominant microorganisms in the SQ and CQ and were the main microorganisms because of their unique functions during the brewing and fermentation process. For instance, *Saccharomyces* served as the main strain for the production of alcohol and other aromatic substances (Chi et al., 2009). *Saccharomycopsis* could produce α-amylase and glucoamylase, contributing to the strong glycosylation ability of the mixture (Chi et al., 2009). *Aspergillus* could produce a large amount of saccharifying hydrolases, which degraded and converted starch into sugars that could be used by bacteria and yeasts and produced proteolytic enzymes that contributed to protein hydrolysis and flavonoid formation (Machida et al., 2008; Gou et al., 2015). *Monascus* not only excreted a variety of hydrolytic enzymes (saccharification enzymes, proteases, esterases, etc.) to improve the brewing quality of Baijiu (Chen et al., 2015), but also produced abundant beneficial metabolites, such as *Monascus* pigments (red, yellow, and orange pigments) (Chen and Li, 2023), gamma-aminobutyric acid (Song et al., 2021), and monacolin K (Zhang et al., 2020), which have antimicrobial functions, lowering blood pressure and blood lipid levels, respectively. *Lactobacillus* could use fermentable sugars to produce lactic acid, acetic acid, and other organic acids to provide *Baijiu* with a unique flavor and regulate the microbial abundance (Xue et al., 2022). Wang and Xu (2019) reported that *Lactobacillus* regulated the composition of other bacteria and yeasts and synthesized flavor compounds to affect the organoleptic properties of liquor. The characteristics of special aromas might be related to unique microorganisms (by Venn diagram) and discriminant microorganisms (by the linear discriminant analysis (LDA) effect size (LEfSe) method), which might also affect the spatial composition and function of microbial communities. For example, *Aspergillus* was identified as a significant discriminant taxon in the SQ, while *Monascus* was identified as a significant discriminant taxon in the CQ based on LEfes analysis (Figure 4). In addition, at the later stage of fermentation, the bacterial richness index of the CQ was greater than that of the SQ and the fungal richness index of the SQ was greater than that of the CQ. These results indicated that the microbial communities in the CQ and SQ demonstrated functional complementarity (Hou et al., 2024).

High-throughput sequencing technology can accurately reveal the species and genetic diversity of microbial communities (Caporaso et al., 2012). However, this the method cannot effectively exclude the impact of dead microorganisms on live microorganisms. Additionally, real microbial strains could not be obtained (Song et al., 2017). Traditional culture-dependent method can obtain a large number of live microorganisms, but it might be affected by the type of culture medium, incubation time, temperature, interactions between species, operation error and so on, making it difficult to accurately reconstruct a large amount of microbial information in the samples (Zheng et al., 2012). The principles of these two methods lead to certain differences in the detection results. These differences need to be analyzed in conjunction with actual situations in order to truly reflect the changes in microbial growth in the GRTQ. For instance, the dynamic changes in the quantity of *Aspergillus*, *Monascus*, and *Lactobacillus* detected by high-throughput sequencing methods (Figures 3C,D) were similar to the results detected via traditional culture-dependent methods in the early stage of fermentation, but there were notable differences in the later stage of fermentation (Figures 5C,E). Due to the increase in temperature and decrease in moisture during the late stage of the fermentation and drying process, some microorganisms died in large numbers. The actual microbial community might be consistent with the results of traditional culture-dependent methods. In other words, traditional cultivation methods with selective culture media could better reflect the actual information. For example, Luo et al. (2023) added 3.3 mL/L acetic acid to YPD medium to achieve effective separation and counting of yeast. Therefore, to better reflect the microbial community information in complex environmental samples, it is necessary to combine two methods for analysis to obtain the most authentic microbial information.

The “green covering” started to appear on Day 2 and peaked on Day 4 from the SQ appearance (Figure 1). These results were basically consistent with the dynamic variation in *Aspergillus* abundance in the SQ from the high-throughput sequencing results (Figure 3C). Through microscopic morphological observation, it was found that the formation of “green-covering” was due to the production of green spores by *Aspergillus* (Figure 8A). The “red heart” began to appear on Day 6, and after the drying process, the red-heart area reached its maximum. Interestingly, this phenomenon was hysteretic compared to the high-throughput sequencing results, which showed that *Monascus* was present in large quantities on Day 3 (Figure 3C). Analyzing the reason, the appearance of *Monascus* did not immediately produce pigments. However, in the later stage of growth, it produced large *Monascus* pigment, which caused the appearance of red color in the core (CQ). However, it is unclear why *A. clavatus* grows externally and *Monascus* prefers to propagate internally. The amounts of “green-covering” and “red-heart” coloring were crucial parameters for evaluating the quality of GTRQ. Excess or low numbers of “green-covering” and “red-heart” led to insufficient microbial diversity and the metabolism of hydrolases. Exploring their growth patterns will enable better guidance for workshop production.

The mechanism of the external growth of *A. clavatus* and the internal reproduction of *Monascus* were analyzed based on the relevant microbial physiology and environmental factors data. The correlation analysis between *Monascus* and environmental indicators was consistent with the research results on the physiology characteristics of *Monascus* (Figure 7A). *Monascus* was positively correlated with temperature (*p* < 0.001). Previous studies demonstrated that *Monascus* required a large amount of water in the early stages of growth (Chen et al., 2015), which was consistent with our finding that as the *Monascus* increased, the water content (*p <* 0.05) decreased (Figure 7A). According to the above results, we speculated that *Monascus* consumed a large amount of water in the CQ at relatively high temperature, resulting in the mass growth of *Monascus* and the production of *Monascus* pigments to further form the red heart. In the early stage of fermentation in the SQ, the moisture and oxygen contents were relatively higher, while the temperature and acidity were lower, which was consistent with the physiological characteristics of *A. clavatus* (Figures 8B,D). Besides, *Aspergillus* grew rapidly and had an advantage in niche competition, thus shaping a “green covering” on the SQ.

## Conclusion

Thus, this study investigated the changes in microbial quantity and community succession during different stages of the fermentation process in the SQ and the CQ. The results showed that there were more bacteria than the fungi in both the SQ and CQ. Furthermore, at the later stage of fermentation, the bacterial richness index of the CQ was greater than that of the SQ, and the fungal richness index of the SQ was greater than that of the CQ. In addition, there were significant differences in the types and quantities of the dominant microorganisms in the CQ and SQ. The dominant microorganisms in the CQ included *Saccharomycopsis*, *Saccharomyces*, *Aspergillus*, *Monascus*, *Lactobacillus*, and *Bacillus*, while those in the SQ were represented by *Saccharomycopsis*, *Aspergillus*, *Monascus*, *Lactobacillus*, *Acetobacter*, and *Weissella*. The study on the correlation between environmental factors (temperature, moisture, and acidity) and dominant microorganisms at the genus level revealed that environmental factors were closely correlated with the reproductive succession of microorganisms. By revealing the physiological characteristics of core microorganisms at different spatial positions of GRTQ, such as *A. clavatus* and *M. purpureus*, as well as their interactions with environmental factors, we elucidated the color formation mechanism behind the phenomenon of “green” outside and “red” inside. This study provides data support for optimizing the production process of GRTQ and offers potential guidance for establishing modern production lines.

## Data availability statement

The datasets presented in this study can be found in online repositories. The names of the repository/repositories and accession number(s) can be found in the article/Supplementary material.

## Author contributions

LiZ: Writing – original draft, Writing – review & editing, Conceptualization, Data curation, Formal analysis, Methodology, Software, Supervision, Validation. LC: Data curation, Investigation, Validation, Writing – original draft. BL: Software, Writing – review & editing. YX: Formal analysis, Funding acquisition, Investigation, Writing – original draft, Writing – review & editing. WD: Conceptualization, Data curation, Methodology, Writing – review & editing, Formal analysis. YL: Methodology, Software, Validation, Writing – original draft. JT: Formal analysis, Investigation, Writing – original draft. GZ: Investigation, Methodology, Validation, Writing – original draft. LeZ: Funding acquisition, Resources, Writing – original draft. SY: Supervision, Validation, Writing – original draft. QY: Formal analysis, Funding acquisition, Investigation, Supervision, Writing – original draft. SC: Funding acquisition, Project administration, Supervision, Validation, Writing – original draft, Writing – review & editing.
