# Supplementary material for: Deciphering the microbial succession and color formation mechanism of “green-covering and red-heart” Guanyin Tuqu
Source: Front Microbiol. 2024 Jul 23;15:1412503. doi: 10.3389/fmicb.2024.1412503 (PMC11300261; doi:10.3389/fmicb.2024.1412503)
Supplement: Supplementary file 1 [file Table_1.DOCX]

Deciphering the microbial succession and color formation mechanism of "green-covering and red-heart" *Guanyin Tuqu*

Liping Zhu^1^, Liang Chen^1^, Bin Lin^1^, Yin Xu^1^, Weiwei Dong^2^, Yijun Lv^1^, Jie Tang^1^, Gang Zhang^1^, Lei Zhang^1^, Shengzhi Yang^1^, Qiang Yang^1^* and Shenxi Chen^1^*

^1^*Hubei Key Laboratory of Quality and Safety of Traditional Chinese Medicine* *and Health Food, Jing Brand Research Institute, Jing Brand Co., Ltd,* *Daye, China*

^2^*Hubei Key Laboratory of Edible Wild Plants Conservation & Utilization, College of Life Sciences,* *Hubei Normal University, Huangshi, China*

**Contents** **Page**

1. Figure S1. Linear discriminant analysis (LDA) effect size (LEfSe) of the central of *Tuqu* (CQ) and the surface of *Tuqu* (SQ) based on species level of fungi (LDA > 3, p < 0.05). 3


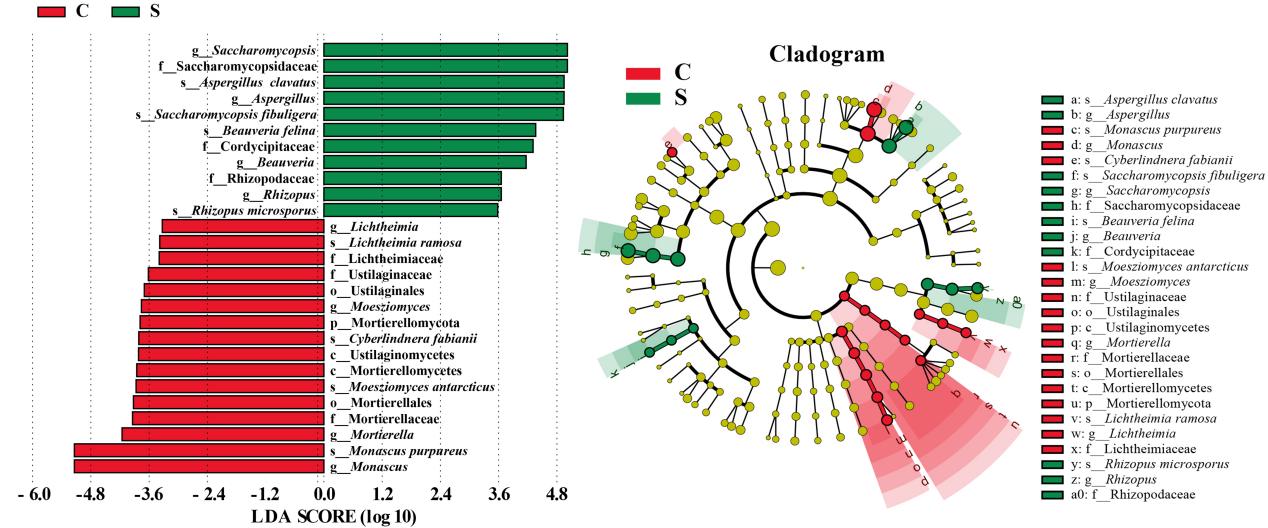


Figure S1. Linear discriminant analysis (LDA) effect size (LEfSe) of the central of *Tuqu* (CQ) and the surface of *Tuqu* (SQ) based on species level of fungi (LDA > 3, p < 0.05). LDA score indicates the level of differentiation between CQ and SQ, and the horizontal bar chart showing discriminant taxa of the fungi. Significant discriminant taxon nodes of CQ and SQ are represented by green and red, respectively, while no discriminant taxon nodes are represented by yellow in the Cladogram. C: Center; S: Surface.
